# Why we dehumanize illegal immigrants: A US mixed-methods study

**DOI:** 10.1371/journal.pone.0257912

**Published:** 2021-10-07

**Authors:** David M. Markowitz, Paul Slovic

**Affiliations:** 1 School of Journalism and Communication, University of Oregon, Eugene, OR, United States of America; 2 Decision Research, Eugene, OR, United States of America; 3 Department of Psychology, University of Oregon, Eugene, OR, United States of America; Iowa State University, UNITED STATES

## Abstract

Dehumanization is a topic of significant interest for academia and society at large. Empirical studies often have people rate the evolved nature of outgroups and prior work suggests immigrants are common victims of less-than-human treatment. Despite existing work that suggests who dehumanizes particular outgroups and who is often dehumanized, the extant literature knows less about *why* people dehumanize outgroups such as immigrants. The current work takes up this opportunity by examining why people dehumanize immigrants said to be illegal and how measurement format affects dehumanization ratings. Participants (*N* = 672) dehumanized such immigrants more if their ratings were made on a slider versus clicking images of hominids, an effect most pronounced for Republicans. Dehumanization was negatively associated with warmth toward illegal immigrants and the perceived unhappiness felt by illegal immigrants from U.S. immigration policies. Finally, most dehumanization is not entirely blatant but instead, captured by virtuous violence and affect as well, suggesting the many ways that dehumanization can manifest as predicted by theory. This work offers a mechanistic account for why people dehumanize immigrants and addresses how survey measurement artifacts (e.g., clicking on images of hominids vs. using a slider) affect dehumanization rates. We discuss how these data extend dehumanization theory and inform empirical research.

## Introduction

Dehumanization is a pervasive social and psychological phenomenon that affects groups of people around the world [1–3]. Immigrants, for example, are viewed as less-than-human compared to ingroups in the US and therefore treated inhumanely through cruel metaphors (e.g., immigrants are animals) [4]. Detainment policies also treat migrants in dehumanizing ways by separating families at country borders [5] and forcing individuals into poorly-resourced detention sites [6]. Who tends to dehumanize immigrants in the US? People who believe immigrants are less-than-human can be characterized by a range of social, psychological, and demographic traits [7], including conservative ideology and the endorsement of seemingly unrelated social harms (e.g., the death penalty for convicted murderers). Even during international crises that should bring groups together (e.g., COVID-19), dehumanization is pervasive and often used as a mechanism to blame outgroups or use them as scapegoats for societal problems [8].

The extant dehumanization literature largely consists of studies describing the outgroups who are dehumanized and who dehumanizes such individuals [9–11]. Less work has focused on the rationales people use when making dehumanization judgments, which are crucial to mitigate or signal dehumanization at its onset. To address this opportunity, our work has two primary aims. First, we complement existing research that typically identifies *who* dehumanizes and *who* is dehumanized by evaluating *why* people dehumanize immigrants. Few studies have explored possible explanations for dehumanization and therefore, we draw on three competing theoretical perspectives (e.g., the less than human hypothesis, virtuous violence, affect heuristic) to motivate our investigation. Our large online experiment also facilitated a second aim, which concerned the impact of measurement format on dehumanization judgments. Typical measures of blatant dehumanization have people rate the humanness of outgroups on a sliding scale of evolving hominids [9]. We tested the prediction that having participants click on an image of evolving hominids will reduce dehumanization relative to making the judgment via a slider, since clicking is a more deliberate and explicit judgment method.

To date, these research aims—understanding (1) why people make dehumanization judgments and (2) how the format of a dehumanization measure affects ratings— have been understudied. Addressing these research goals will clarify how we might better identify dehumanizers, understand their motivations, and evaluate whether the measurement of dehumanization impacts the judgment of perceived outgroups. We therefore offer methodological contributions by describing how survey artifacts impact dehumanization judgments and theoretical contributions by assessing the relative importance of rationales that explain why people tend to dehumanize immigrants.

### Competing views of dehumanization

Dehumanization can develop as tacit or overt forms of aggression and hatred toward an outgroup by members of an ingroup, where ingroup members believe such “others” do not have the same level of humanity (e.g., emotions, feelings, experiences) [7, 9, 10]. Tacit forms of dehumanization include infrahumanization [10, 12, 13], which describes ingroup members who believe that an outgroup cannot experience secondary emotions (e.g., those that are uniquely human, such as embarrassment) and therefore treat them differently. Blatant forms of dehumanization include insensitive metaphors and labels to associate with an outgroup (e.g., calling COVID-19 the “China Virus” or “Wuhan Virus”), furthering the belief that certain groups are less evolved, less human, or less deserving of humanity than others [9, 14]. Blatant dehumanization, which is the primary focus of the current work, is common for many outgroups (as rated by American and British participants) and is often measured by participants rating groups on an evolution scale [9]. Therefore, from prior work, it is clear that many groups are dehumanized, and dehumanization perpetrators are characterized by a range of traits (e.g., demographics, policy views). Why such individuals dehumanize, however, is less clear and represents the primary interest of this paper.

People can perceive outgroups as less than human for many reasons. Some people might genuinely believe that outgroups such as immigrants are unevolved, while others might dislike immigrants because they come from different cultures. Both perspectives are equivalent in traditional conceptualizations of dehumanization (see [15]), though they are clearly associated with different social and psychological dynamics. The former example denies the humanity of immigrants and is blatantly dehumanizing while the latter example is associated with heightened negative affect, but not the denial of a group’s humanity.

To disentangle how people feel toward immigrants as a reflection of their humanness judgments, we draw on prior literature that identifies dominant rationales for dehumanization. Specifically, we test the *less than human hypothesis* [9, 14], the *virtuous violence hypothesis* [15, 16], and the *affect heuristic hypothesis* [17]. While the differences between hypotheses are addressed below, there are several commonalities worthy of discussion. First, the hypotheses are largely based on psychological theory [10, 12]. According to these perspectives, people use psychological processes and systems to make judgments about outgroups, and such judgments can affect a host of psychological dynamics (e.g., how people feel toward a target group). Instead of dehumanization being a philosophical problem, it represents and reflects the internal processing of intergroup relations. Second, the outcomes of dehumanization according to these perspectives are almost always negative for the victims. Dehumanizers might feel justified or righteous for their views, but dehumanized outgroups suffer greatly as a result of a dehumanizing act. Finally, the hypotheses and their associated dehumanization unfold in diverse settings. As the proceeding evidence describes, dehumanization occurs in education, medicine, hypothetical wartime scenarios, and intergroup relations. Therefore, dehumanization is a rich psychological process that spans groups and settings.

The *less than human hypothesis* argues that people believe particular outgroups are indeed unevolved; their humanity and capacity for human feelings is less than an ingroup. Consistent with this perspective, people often believe that Americans are more evolved than Mexican immigrants [9] and blatant dehumanization goes beyond prejudice or hatred toward particular outgroups. Discrimination of students by Hungarian teachers, for example, was predicted by blatant dehumanization after controlling for affect via a feeling thermometer and subtle dehumanization via the denial of secondary emotions [14]. Therefore, according to the *less than human hypothesis*, people treat immigrants harshly (e.g., more discrimination, harsher jail sentencing if convicted of illegal activity) because they believe their humanness is inferior to other groups and they are incapable of thinking and feeling like other humans [7, 9, 10, 14, 18].

The *virtuous violence hypothesis* argues that people punish, inflict violence on others, and dehumanize them because they believe it is the right thing to do [19]. As a result, members of an ingroup believe the outgroup deserves harsh treatment, pain, or suffering [16]. Violence and associated dehumanization, therefore, is intentional and facilitated by a need to achieve instrumental violence (e.g., violence used to obtain a secondary goal). An ingroup (e.g., Americans) typically believes that it is their right and purpose to harm others who are human and capable of agency (e.g., immigrants) to help pursue other instrumental goals or tasks [15]. A similar argument has been proposed in other settings, such as the approval of war crimes when the war was believed to be justified [20]. Virtuous violence can therefore become a pathway for dehumanization. People may dehumanize illegal immigrants by acknowledging their humanity, but also suggest they deserved worse treatment than an ingroup for a range of (prejudiced) reasons (e.g., lower intelligence, lower morals).

A third possibility, the *affect heuristic hypothesis*, argues that people use their feelings to judge how much they like or dislike a particular group [17]. Emotions, therefore, link to how people think and feel about ingroups and outgroups [11]. Prior work suggests people who overestimate the threat posed by immigrants tend to hold negative views toward them [21, 22] and people who have more positive attitudes toward alienated outgroups (e.g., the homeless) tend to rate them as more human over time [23]. According to the affect heuristic, more familiar groups are perceived positively compared to less familiar groups. Unfamiliar groups, such as immigrants or foreigners, should therefore be associated with increased rates of negative affect and decreased warmth [24]. Here, dehumanization starts with and is reflected by feelings.

These three competing perspectives provide an opportunity to explore why people might dehumanize immigrants, a group who receives poor treatment globally. This empirical question is important as the current dehumanization literature often fails to deeply understand *why* people dehumanize [10]. We do not offer a formal prediction about rates of dehumanization according to each theoretical perspective, but instead explore how dehumanization manifests in line with these ideas. As described below, we also investigate how dehumanization is linked to social, psychological, and demographic measures from prior work to facilitate a replication and extension effort [7]. Replications are crucial to test the validity of empirical findings [25] and extensions offer new and complementary ways to conceptualize scientific problems. In our effort to understand why people dehumanize immigrants as aligned with theory (e.g., less than human hypothesis, virtuous violence, affect heuristic), our exploratory measures (e.g., perceived unhappiness felt by immigrants) also helped to explain why dehumanization might persist.

### The impact of measurement format

Our second interest examines how measurement format impacts blatant dehumanization ratings. Most research using the Ascent of Man evolution scale has participants use sliders to make their dehumanization judgments [7, 9, 14]. We examine how moving a slider across a numbered line versus clicking on the image of evolving hominids impacts ratings of illegal immigrants. Survey methodology research argues sliders produce different response times than clicking radio buttons [26], and here we suggest people might engage in more deliberate processing when clicking to judge an outgroup’s humanness versus a slider. Such differences are associated, in principle, with System 1 (e.g., automatic, intuitive, fast thinking) versus System 2 (e.g., analytical, deliberate, slow thinking) modes of thought [27]. Using a slider to indicate blatant dehumanization might produce a less intentional, relative response compared to clicking on the image of evolving hominids, which would force respondents to think more deeply and deliberately about their decision to rate illegal immigrants as less than fully human. We therefore propose:

H_1_: Participants who use a slider will dehumanize illegal immigrants more than participants who click on images of evolving hominids.

We also explored how identifying or “calling out” one’s dehumanization might impact their subsequent dehumanization ratings. If participants dehumanized (e.g., rated illegal immigrants as less than fully evolved across measurement formats), we asked them why they dehumanized and gave them an opportunity to change their rating. The rating changes were not evaluated in this manuscript, however, to remain focused with more central measures.

## Method

We estimated a small effect size (Cohen’s *d* = 0.25) with 80% power (α = 0.05, two-tailed) to evaluate how the measurement format (slider vs. clicking) affected dehumanization ratings. This required 506 participants to detect an effect across two conditions of our study. We oversampled recruitment (700 participants), ensuring a large and even distribution of self-identifying Democrats and Republicans since prior evidence suggests political identity is a strong predictor of dehumanization [7, 11, 28]. Based on criteria from prior work [7], we also removed any participants with less than or equal to 15 words in their writing output (*n* = 28; see below). Our final sample contained 672 participants, collected on March 9–10, 2020, and the study received ethics approval from Decision Research.

### Participants

We recruited participants from Prolific and they were paid for their time. The average participant was 35.88 years old (*SD* = 13.86 y). Participants were mostly female (*n* = 354; male, *n* = 310; other, *n* = 8), and we achieved an even distribution of self-identifying Democrats (*n* = 312; 46.4%) and Republicans (*n* = 304; 45.2%). Thirty-five participants also self-identified as independents, fourteen self-identified as undeclared, and seven self-identified as “other” for political identity. We collapsed self-identified independents, undeclared, or other into a single level called “other.” Random assignment across our measurement format factor (slider vs. clicking) was indeed successful as gender, [χ^2^(2) = 3.72, *p* = .155], age, [*t*(669) = .03, *p* = .980], and political identity, [χ^2^(2) = 2.37, *p* = .305], were not significantly different across conditions.

### Procedure

This study’s procedure closely followed other work to facilitate a replication effort [7], but contained some differences that aligned with our extension effort as well.

After obtaining informed consent, participants were presented with a scenario and told that an immigrant was caught entering the United States by illegally crossing the southern border. They were also told that the immigrant might face jail time if convicted. Participants then indicated the amount of jail time the immigrant should be sentenced to (henceforth called the jail time scale): (1) None, (2) Days, (3) Weeks, (4) Months, (5) Years, and (6) Life in jail. Next, participants wrote into an essay text box their thoughts and feelings about the jail time judgment based on the following instructions (henceforth called the general prompt):

*Now, tell us what you are thinking and feeling about the judgment you made. You can discuss why you believe this punishment should be granted, how you feel about immigration, your thoughts on immigrants, U.S. policies about separating children from their parents until their immigration case can be adjudicated, requiring asylum seekers to first try for asylum in another country, or other related topics that may come to mind. Please be specific and detailed*.

Participants then responded to a measure of blatant dehumanization (e.g., the Ascent of Man) [9] via slider (*n* = 333) or clicking format (*n* = 339) depending on their randomized condition. Upon submitting their response, we added a display logic to our survey that allowed us to understand why people dehumanized illegal immigrants. If any participant rated illegal immigrants as less than human (e.g., anything other than the farthest right response; total *n* = 125/672; 18.6%), we asked them about their dehumanization via the following instructions (henceforth called the rationale prompt):

*You just made a judgment that illegal immigrants are less than human. Why did you make this judgment and what led you to decide that illegal immigrants are less than human?*

An essay text box appeared and only participants who dehumanized wrote about their rationale for rating illegal immigrants as less than human. Finally, participants responded to measures including perceived warmth toward immigrants [24], perceived unhappiness felt by immigrants with US immigration policies, social closeness toward immigrants [29], the endorsement of social harms, adverse childhood experiences [30], and demographics.

### Measures: Less than human hypothesis

#### Blatant dehumanization

We used the Ascent of Man (AOM) as a measure of blatant dehumanization [9], where only the right-most image is a fully evolved human.

Our experimental manipulation had participants make blatant dehumanization ratings on different formats: a slider or a clicking format. This allowed us to evaluate how rates of blatant dehumanization might be related to measurement format. Those randomly assigned to the slider condition rated the evolved nature of illegal immigrants using an 8-point slider (1: unevolved, 8: fully evolved) [7]. Those randomly assigned to the clicking condition clicked on one of five images in the AOM scale, from (1) not evolved to (5) fully evolved. We outlined each image on the evolution scale using the “hot spot” feature of Qualtrics to have participants click and submit responses to only one figure (see S1 Fig in S1 File).

Note, since the slider and clicking questions were on different scales, we rescaled the responses by dividing each participant score by the highest value for each measure. For example, all slider scores were divided by 8 and all clicking scores were divided by 5. Each score represents a percent less-than-human score and had an upper-bound of 100%, or fully evolved.

#### Social closeness

We evaluated how close people felt toward illegal immigrants using the Inclusion of the Other in the Self (IOS) scale [29], since prior work suggests people who blatantly dehumanize immigrants tend to feel more social distance toward them [7]. This measure has participants judge their social closeness to a group based on (1) non-overlapping to (5) almost fully overlapping circles. We measured closeness to illegal immigrants, United States citizens, Democrats, and Republicans. Due to space considerations, we only discuss responses to the illegal immigrant item of the IOS scale.

### Measures: Virtuous violence hypothesis

#### Why I dehumanize

If participants dehumanized illegal immigrants on the AOM scale (e.g., any response less than fully evolved), they responded to four questions after the rationale prompt. All statements were on 7-point scales from strongly disagree to strongly agree: (1) “Illegal immigrants need to be punished to teach them a lesson,” (2) “I feel badly when I see illegal immigrants punished,” (3) “Illegal immigrants are inferior to other people,” and (4) “Illegal immigrants are unfamiliar to me.” The second question was only associated with political ideology and reported in Table 1.

**Table 1:** Key results across measurement type and political parties.

		Clicking		Slider				
Panel 1	Measure	*M*	*SD*	*M*	*SD*	*t*	*p*	*d*
Less than human	Ascent of man (%)	95.8	13.4	91.1	19.4	3.58	< .001	0.28
		Democrat		Republican				
Panel 2	Measure	*M*	*SD*	*M*	*SD*	*t*	*p*	*d*
Less than human	Ascent of man (%)	97.9	9.5	88.4	21.4	7.08	< .001	0.57
	Jail time scale	2.00	1.10	3.37	1.19	-14.85	< .001	1.20
	Inclusion of the Other in the Self	2.54	1.23	1.56	0.91	11.37	< .001	0.91
Affect heuristic	Warmth	70.67	24.30	28.19	25.92	20.93	< .001	1.69
	Unhappiness index	4.73	0.56	3.72	1.05	14.68	< .001	1.20
	Unhappiness: Family separation	4.86	0.52	4.22	1.03	9.67	< .001	0.78
	Unhappiness: Discrimination	4.61	0.74	3.54	1.22	13.05	< .001	1.06
	Unhappiness: Harsh conditions	4.71	0.65	3.40	1.36	15.14	< .001	1.23
Other measures	Harms index	0.46	0.72	2.39	0.77	-32.02	< .001	2.59
	Vulnerability index	2.19	0.55	1.93	0.53	6.04	< .001	0.48
		Dehumanizers		Non-dehumanizers				
Panel 3	Measure	*M*	*SD*	*M*	*SD*	*t*	*p*	*d*
Less than human	Jail time scale	3.52	1.26	2.48	1.27	8.32	< .001	0.82
	Inclusion of the Other in the Self	1.45	0.82	2.22	1.21	-8.57	< .001	0.74
Affect heuristic	Warmth	25.16	26.74	56.09	31.06	-11.26	< .001	1.07
	Unhappiness index	3.49	1.17	4.41	0.84	-8.35	< .001	0.90
	Unhappiness: Family separation	3.91	1.17	4.70	0.71	-7.25	< .001	0.82
	Unhappiness: Discrimination	3.33	1.28	4.26	1.03	-7.62	< .001	0.80
	Unhappiness: Harsh conditions	3.22	1.42	4.27	1.10	-7.73	< .001	0.83
Other measures	Harms index	2.21	0.92	1.22	1.19	10.25	< .001	0.93
	Vulnerability index	1.98	0.55	2.08	0.54	-1.90	.057	0.18
		Democrat dehumanizers		Republican dehumanizers		Other dehumanizers		
Panel 4	Measure	Agree	Disagree	Agree	Disagree	Agree	Disagree	φ
Virtuous violence	I dehumanize to teach immigrants a lesson	43.5%	56.5%	86.2%	13.8%	66.7%	33.3%	0.404***
	I feel bad when immigrants are punished	70.0%	30.0%	23.5%	76.5%	42.9%	57.1%	0.384***
	Immigrants are inferior to me	14.3%	85.7%	51.2%	48.8%	50.0%	50.0%	0.295**
	Immigrants are unfamiliar to me	31.8%	68.2%	52.4%	47.6%	60.0%	40.0%	0.172

*Note*.

*** *p* < .001

** *p* < .01 for chi-square tests.

### Measures: Affect heuristic hypothesis

#### Affect

Prior work by Sagan and Valentino (2017) suggests warmth toward an outgroup is inversely related to justified violence toward them. We examined this effect with illegal immigrants and presented participants with the following prompt via a 0–100 slider:

*Please rate your feelings toward illegal immigrants, with one hundred meaning a very warm, favorable feeling, zero meaning a very cold, unfavorable feeling, and fifty meaning not particularly warm or cold. You can use any number from zero to one hundred. The higher the number the more favorable your feelings are toward illegal immigrants*.

#### Unhappiness felt by immigrants

Recall, prior research suggests that a form of dehumanization is the denial of an outgroup’s secondary emotions (e.g., nostalgia), a concept called *infrahumanization* [10]. While infrahumanization refers to specific secondary emotions denied for an outgroup, we were more concerned with the feelings of our participants, how they might indicate one’s tendency to dehumanize, and how they perceived the level of unhappiness experienced by illegal immigrants across different circumstances. All participants rated the perceived unhappiness felt by illegal immigrants related to family separation, discrimination, and harsh and unsafe living conditions while waiting for judicial hearings. We used these three items to connect to the prior warmth measure and the *affect heuristic hypothesis*, which suggests that people who are less warm to illegal immigrants will want to punish them more. Each item was measured on a scale of (1) Not at all to (5) A great deal. The items were statistically reliable as a composite, (Cronbach’s α = 0.88), and therefore averaged into an unhappiness index.

### Other measures connected to dehumanization

The following measures further assisted with our replication and extension effort of prior research: perceived vulnerability in society, the endorsement of social harms, adverse childhood experiences, and an assessment of why participants believe others might dehumanize. We connected these measures to blatant dehumanization.

#### Vulnerability

Consistent with prior dehumanization research [7], we assessed the degree to which people felt personally vulnerable in society with six questions, which were combined into a vulnerability index (Cronbach’s α = 0.73). All items are located in the online supplement out of space considerations.

#### Social harms

The endorsement of social harms significantly associates with dehumanization toward immigrants [7]. We asked participants for their support (scored as 1) or disapproval (scored as 0) of three social harms: gun ownership, immigrant raids, the death penalty. Responses were summed to form an index (Cronbach’s α = 0.74) and high scores suggest the endorsement of more social harms than low scores.

#### Adverse childhood experiences

We evaluated whether adverse childhood experiences (e.g., parental divorce, substance abuse by a parent, sexual assault) related to dehumanization, an effect also obtained in prior work [7]. We used the Adverse Childhood Experiences (ACE) scale (Cronbach’s α = 0.78), which includes ten items ranging from divorce to mental health issues among family members during childhood. Please see the online supplement for these data.

#### Why others dehumanize

We told participants that “Some of our survey participants rated illegal immigrants as less than fully evolved human beings. Please rate how much you agree with reasons why people would rate illegal immigrants as less than fully evolved.” We asked three questions on a scale of (1) Strongly disagree, to (5) Strongly agree: (a) “They truly believe these immigrants are less than human,” (b) “They simply don’t like illegal immigrants,” and (c) “This allows them to feel less guilty about the harsh treatment immigrants are receiving.” These questions were evaluated independently and connected dehumanization with perceptions of others’ behavior.

### Automated text analysis

We used two tools to evaluate text data from the general prompt (What are you thinking about the punishment judgment you just made?) and the rationale prompt (Why did you judge illegal immigrants as less than human?). The first tool, Linguistic Inquiry and Word Count (LIWC) [31], uses a dictionary-based approach to count words as a percent of the total word count for each input text. LIWC’s internal dictionary contains a range of categories across social, psychological, and part of speech dimensions. We evaluated five dimensions often assessed in studies of how people dehumanize immigrants [7]: impersonal pronouns (e.g., *it*, *anyone*), emotion words (e.g., *happy*, *terror*), positive affect (e.g., *exciting*, *amazing*), negative affect (e.g., *hate*, *awful*), and power words (e.g., *dominant*, *superior*). Dehumanizers and those who would sentence immigrants to harsh jail time tend to distance themselves from the perceived outgroup by making fewer personal references to them, their verbal output is more emotional, and they discuss immigration issues from a position of power compared to non-dehumanizers.

The second tool, the Meaning Extraction Helper (MEH) [32], was applied just to the rationale prompt data and automates the Meaning Extraction Method. This technique removes function words (e.g., pronouns, articles) from a text and allows content words (e.g., nouns, verbs) to cluster statistically [33, 34]. MEH applies a score of 1 to word if it is present in a person’s text and a score of 0 if it is absent. Then, using Principal Component Analysis (PCA) with varimax rotation, themes can emerge from content words. A fixed number of factors (*n* = 5) were retained in the PCA after considering scree plot evidence and thematic interpretability. Items were retained if loadings were ≥ |.30| after rotation and if they appeared in at least 3% of texts.

## Results

Descriptive statistics for key variables are located in S1 Table in S1 File. We also provide a full correlation matrix of our main variables (S2 Table in S1 File) and tables separated by clicking (S3 Table in S1 File) and slider conditions (S4 Table in S1 File). Correlations between variables were largely in the same direction across conditions, though stronger relationships were often observed for those in the slider condition compared to the clicking condition. Effects in the main text are organized by dehumanization hypothesis. Data are available on the Open Science Framework (https://osf.io/cjnu5/).

### Less than human hypothesis

**Fig 1:**
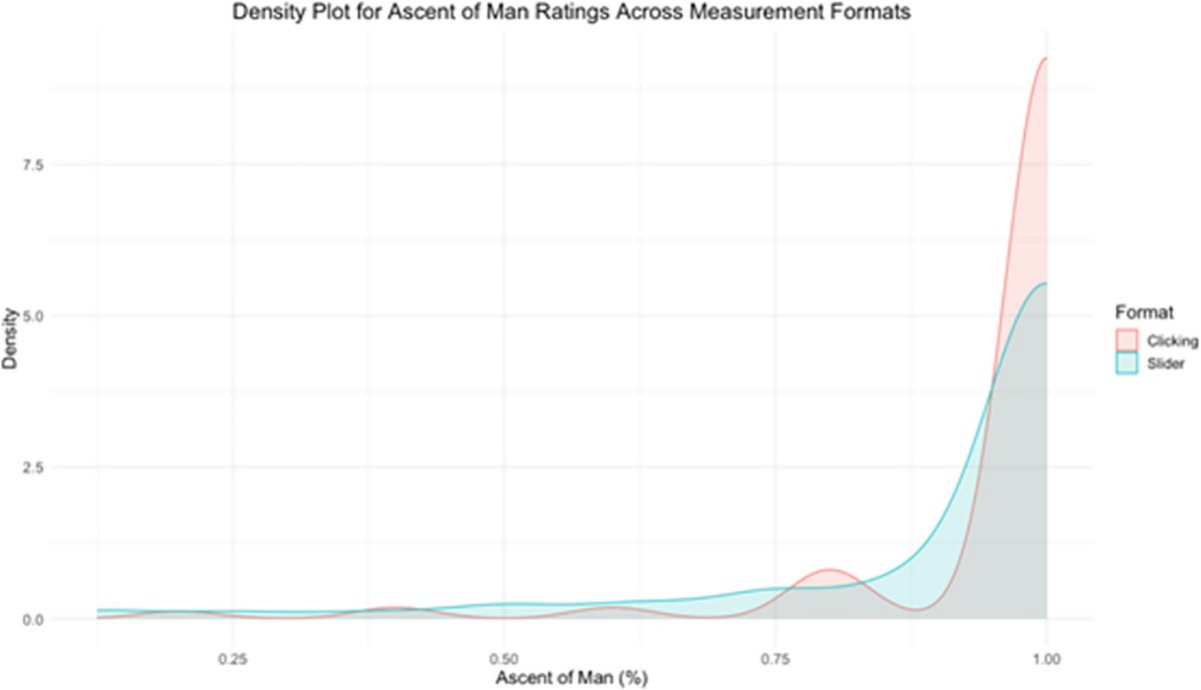
Distribution of dehumanization ratings across measurement formats. This figure is a density plot for Ascent of Man ratings across measurement formats.

Scores on the AOM were transformed into percentages and lower scores indicate more dehumanization. As the top panel of Table 1 reveals, the level of dehumanization was greater for those who used the slider compared to those who clicked on Ascent of Man images [Welch’s *t*(590.72) = 3.58, *p* < .001, Cohen’s *d* = 0.28]. To evaluate the distribution of responses across measurement formats, please see the density plot in Fig 1.

Since measurement formats were on different scales, however, it was also important to evaluate if the number of participants who made less-than-fully-human ratings were significantly different across conditions. The number of participants who dehumanized in the slider condition (*n* = 83/333; 24.9%) was nearly double the number of participants who dehumanized in the clicking condition (*n* = 42/339; 12.4%), [χ^2^(1) = 17.43, *p* < .001, φ_c_ = .161]. Together, these findings support H_1_.

**Fig 2:**
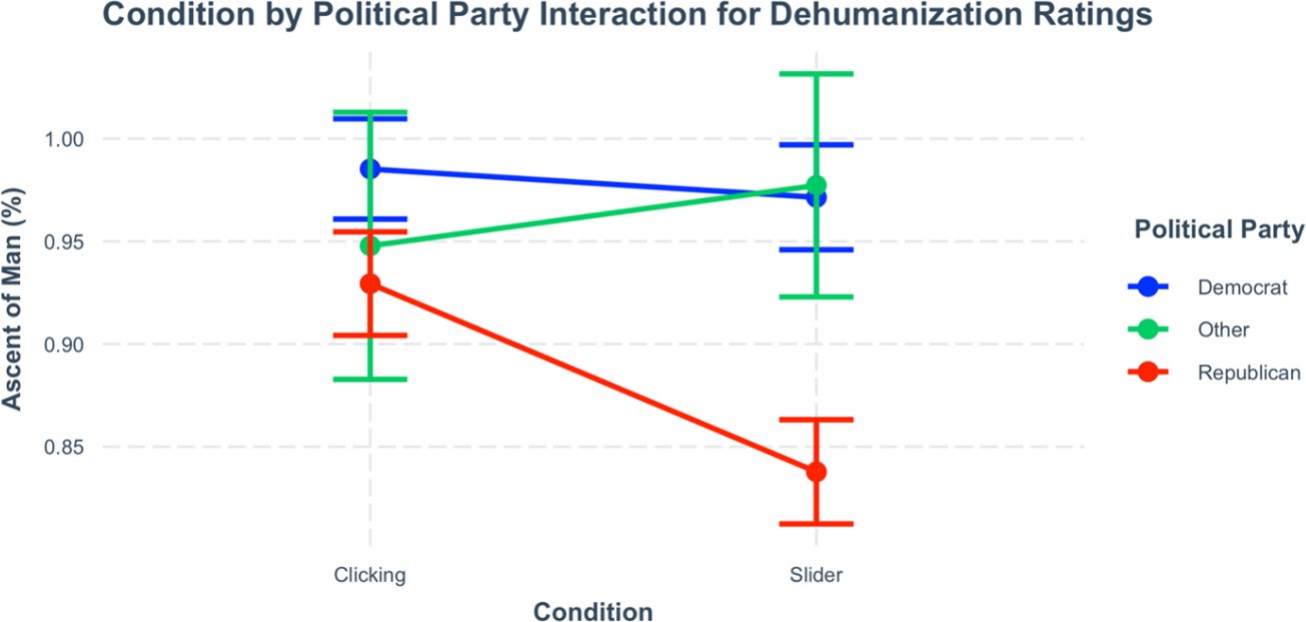
Condition by political party interaction. Political party X condition interaction effect demonstrating that measurement format affects Republicans relative to other political parties. Error bars are 95% confidence intervals.

Replicating prior work, Democrats dehumanized illegal immigrants less than Republicans, *p* < .001 (the second panel of Table 1). However, a significant Measurement format (clicking vs. slider) X Political party (Democrat, Republican, other) interaction effect also emerged, [*F*(2, 666) = 6.27, *p* = .002]. Republicans tended to rate illegal immigrants as more evolved when clicking on images (*M* = 92.9%, *SE* = 1.3%) compared to using a slider [(*M* = 83.8%, *SE* = 1.3%), *p* < .001, *Bonferroni*-corrected; see Fig 2]. Other post-hoc tests were not significant, *p*s > .444. This effect suggests measurement format impacts dehumanization ratings especially for those who are more typical dehumanizers, such as Republicans.

The third panel of Table 1 reveals the patterns that differentiate dehumanizers (e.g., those who rated illegal immigrants as less than fully human across measurement formats) from non-dehumanizers (e.g., those who rated illegal immigrants as fully human across measurement formats). The strongest effect was ratings of warmth (Cohen’s *d* = 1.07), where non-dehumanizers tended to rate their feelings toward illegal immigrants as warmer than dehumanizers. In general, the effect sizes separating dehumanizers from non-dehumanizers were large (average Cohen’s *d* = 0.79) and in the expected direction. With respect to warmth and the endorsement of social harms, these effects were larger and more adverse toward immigrants than the dehumanization measure. While dehumanization is strong and linked to harm, the data suggest Republican ideology is more hostile than dehumanization.

### Virtuous violence hypothesis

People who would sentence an illegal immigrant to more jail time also wrote from a position of power (*r* = .141, *p* < .001; S1 Table in S1 File). These data are therefore a positive replication of Markowitz and Slovic [7].

#### Why I dehumanize

People would send an illegal immigrant to more jail time to teach them a lesson (*r* = .571, *p* < .001) and because they believe immigrants are inferior to others, (*r* = .364, *p* < .001). Similar patterns emerged when replacing jail time with the AOM measure.

We also regressed the jail time scale on the three rationales for participant dehumanization to understand the potentially virtuous nature of rating immigrants as less than human (*R*^2^ = 0.34). Since participants offered a response to each of the rationales, we evaluated how they might independently predict dehumanization rates. The rationales included: to teach immigrants a lesson (*B* = 0.36, *SE* = 0.06, *t* = 6.21, *p* < .001); because immigrants are inferior to other people (*B* = 0.10, *SE* = 0.06, *t* = 1.76, *p* = .081) and immigrants are unfamiliar to me, (*B* = -0.03, *SE* = 0.05, *t* = -0.61, *p* = .545). Replacing the jail time scale with the AOM (*R*^2^ = 0.28), revealed similar effects: people tend to dehumanize illegal immigrants to teach them a lesson (*B* = -0.04, *SE* = 0.01, *t* = -3.49, *p* = .001) and because they believe illegal immigrants are inferior to other people, (*B* = -0.04, *SE* = 0.01, *t* = -3.71, *p* < .001). People dehumanize marginally less if they rate illegal immigrants as unfamiliar to them, (*B* = 0.02, *SE* = 0.01, *t* = 1.91, *p* = .059). The data suggest dehumanization is often rooted in an ingroup (e.g., Americans) believing that an outgroup (e.g., immigrants) can feel and deserves punishment (e.g., to teach them a lesson); they are less often associated with ingroups believing that outgroups are unfamiliar or inferior.

We also evaluated dehumanization rationales by political party (e.g., self-identified Democrats, Republicans, and other). To obtain simple ratings of agreement or disagreement with each statement, we transformed the scale responses into dichotomous variables (scores 1–3 = disagree, scores 5–7 = agree), excluding those who answered at the midpoint of each 7-point scale. The results suggest that ratings on these measures were indeed polarized by political affiliation (see the fourth panel of Table 1). The most politically divisive rationale was “I dehumanize to teach immigrants a lesson”: over 85% of Republicans agreed with this statement and therefore believed immigrants deserve punishment. While more Democrats disagreed instead of agreed with the idea that they dehumanized to each immigrants a lesson, agreement was still relatively high (S1 Table in S1 File). Perhaps this effect emerged because our prompt described illegal actions, which might be perceived as deserving some punishment.

Finally, over 75% of Republicans reported they do not feel bad when immigrants are punished, whereas 70% of Democrats felt the opposite. Over 85% of Democrats disagreed that immigrants are inferior to them and just over 50% of Republicans agreed that immigrants are inferior. Note, we obtained substantively equivalent patterns of results if scale measures were used to compare mean responses across political parties.

### Affect heuristic hypothesis

Consistent with prior work [24], people would sentence illegal immigrants to more jail time if they felt colder toward them (*r* = -.603, *p* < .001). Dehumanizers would sentence illegal immigrants to more jail time if they believed illegal immigrants experienced less unhappiness due to harsh conditions (*r* = -.425, *p* < .001), discrimination (*r* = -.398, *p* < .001), and family separation (*r* = -.369, *p* < .001). All unhappiness measures predicted jail time for illegal immigrants in a multiple regression model (*R*^2^ = .198), *t*s > -1.94, *p*s < .053. It is noteworthy, however, that the average level of perceived unhappiness was above the midpoint for both Democrats and Republicans (S1 Table in S1 File), suggesting people generally believe illegal immigrants perceive high levels of unhappiness from US immigration policies.

Harsh jail time punishment was associated with reduced warmth and the belief that immigrants have a reduced ability to feel unhappiness. To tease apart these effects independently, we regressed the jail time scale on warmth (*B* = -0.02, *SE* = 0.001, *t* = -14.06, *p* < .001) and the unhappiness index, (*B* = -0.23, *SE* = 0.05, *t* = -4.64, *p* < .001). Both measures significantly predicted jail time (*R*^2^ = .38), though warmth was more strongly associated with jail time than unhappiness. Therefore, emotion is also crucial in dehumanization judgments.

### Exploratory measures

#### Why others dehumanize

We regressed the jail time scale on three reasons why others might dehumanize illegal immigrants (*R*^2^ = 0.077): others believe they are indeed less than human, they dislike illegal immigrants, and they dehumanize to feel less guilty about harsh treatment. Two rationales were significantly related to the jail time scale: others dislike illegal immigrants (*B* = 0.14, *SE* = .05, *t* = 2.84, *p* = .005) and others want to feel less guilty about the harsh treatment illegal immigrants receive (*B* = -0.28, *SE* = .04, *t* = -6.80, *p* < .001). The less-than-human belief by others was unrelated to jail time, (*B* = -0.06, *SE* = .04, *t* = -1.52, *p* = .129).

We also regressed the AOM on the three proposed rationales for why others dehumanize, which accounted for nearly 10% of explained variance. Two rationales were significantly related to the AOM: the belief that illegal immigrants are less than human, (*B* = -0.02, *SE* = .01, *t* = -4.64, *p* < .001), and to feel less guilty about harsh treatment, (*B* = 0.04, *SE* = .01, *t* = 7.90, *p* < .001). Dislike was not related to the AOM, (*B* = -0.002, *SE* = .01, *t* = -0.34, *p* = .737).

### Thematic review of dehumanization rationales

Using the rationale prompt data analyzed with MEH, we examined the reasons why people dehumanized illegal immigrants. The data in S5 Table in S1 File suggests the rationales focused on the topics of illegality, criminality, and law breaking. Therefore, dehumanizers often cite policy and rules as a reason for their blatant disregard for the humanity of immigrants, and not simple dislike.

#### Qualitative review

We also qualitatively reviewed all rationales in search of trends related to the *less than human hypothesis*, *virtuous violence hypothesis*, and *affect heuristic hypothesis*. Upon review, we added another category called *recantations*, which described participants who reconsidered their rating, those who made mistakes in their judgment, or those who misinterpreted the AOM. The qualitative codebook is available in the Supporting information.

Two independent coders rated each rationale for their placement in one of four categories and achieved good agreement (Cohen’s κ = .794, *p* < .001). We used this approach to identify the dominant theme of each rationale and coders resolved discrepancies after discussion. Note, two responses did not fit one of the four categories and were therefore excluded from the rationale dataset (final *n* = 123). Table 2 outlines the number of cases per category and example rationales.

**Table 2:** Rationales for dehumanization across hypotheses and recantations.

Rationale	*n* (%)	Examples
Less than human hypothesis	49 (39.8%)	“Rapists and murders are not human.”
		“They don’t seem to have a moral compass like other humans do. They disregard laws that others would never break. They come to the US in order to get free healthcare. They’re only thinking of themselves and not how that affects our country and our citizens. And they don’t care! How do people think like that? How do they live with themselves?”
Virtuous violence hypothesis	35 (28.5%)	“I believe they are human, but they are unethical in the way that they conduct themselves. I don’t think they are any less intelligent, but their lives are not as modern and sophisticated as Americans. Most of them don’t know how to make it when they get to America and often end up working for slave wages illegally and taking jobs away from others.”
		“I didn’t necessarily mean less than human. Just less civilized and educated.”
Recantations	22 (17.9%)	“I guess I didn’t understand the question, I believe everyone is equal, unless their actions prove otherwise.”
		“I guess I didn’t realize that I was saying anyone was less than human. However I put Americans on the same level and generally would say the same about anyone else. When we stop with hatred, violence, law breaking and killing each other, then I would say we are fully human. We are fully human when we ALL stop acting like animals”
Affect heuristic hypothesis	17 (13.8%)	“I am not fond of illegal immigrants at all. They make our country worse and cause more bad than good. Enter the country legally like most other people have to in other countries. The entitlement of illegal immigrants is absolutely ridiculous.”
		“Honestly? Because I’m racist. I lived with a family of illegal immigrants when I was in a pinch a few years ago and came to absolutely hate their guts because of their nasty, entitled attitudes. My horrible relationship with them has severely damaged my perception of both specifically Mexicans and illegal immigrants in general.”

*Note*. Categories are sorted in descending frequency. Two participants were excluded from the qualitative review since they provided responses outside the four categories (final *n* = 123).

Perceiving illegal immigrants as less than human was the most prevalent rationale (39.8%). People openly admitted their hatred for illegal immigrants by using metaphors (e.g., “murderers”, “animals”) and argued that “anyone who decides that society’s laws no longer applies to them has given up their humanity.”

Many rationales (28.5%) were also consistent with the virtuous violence hypothesis. Participants often acknowledged the humanity of illegal immigrants but suggested why they deserved harsh treatment. People suggested illegal immigrants are human but lack intelligence, make poor decisions, are “less civilized and educated.”

People who recanted their original dehumanization rating (17.9%) offered several explanations. They often misunderstood the figure (*n* = 8; “I guess I didn’t realize that I was saying anyone was less than human.”), made a mistake (*n* = 3; “Wrong judgment”), or rated illegal immigrants and Americans at the same level on the AOM (*n* = 11; “I rated illegal immigrants the same as Americans.”). We suspect these are genuine recantations—people are not dehumanizers and made an erroneous dehumanization rating—or participants felt bad about being called a dehumanizer and made a socially desirable response. It is also noteworthy that 4.5 times more recantations occurred in the slider (*n* = 18) versus the clicking condition (*n* = 4).

Finally, participants who relied on their feelings to judge illegal immigrants or reported on negative personal experiences with immigrants often wrote in terms of dislike (13.8% of those who dehumanized). These individuals relied on emotions as a shortcut to dehumanize (“I don’t like that they disrespect the laws of our country and enter illegally.”) and often perceived illegal immigrants as undesirable members of society (see [35]).

#### Additional quantitative evidence

We used the four rationales as levels of an independent variable to predict key outcomes from our study. S6 Table in S1 File suggests our qualitative coding also revealed systematic quantitative patterns about dehumanization.

Recantations—those who did not understand the Ascent of Man, made a mistake, or rated immigrants and Americans equally on that scale—and those in the virtuous violence category had statistically equivalent AOM ratings. That is, *Bonferroni*-corrections for multiple comparisons revealed that people who recanted and those in the virtuous violence category rated illegal immigrants with statistically equivalent levels of humanness. Those with a virtuous violence rationale, however, rated illegal immigrants as significantly more human than those with less than human or affective rationales. This result is reasonable because virtuous violence, by definition, still acknowledges the human nature of an outgroup but ingroup members believe that they deserve to suffer [16]. People who recanted also rated illegal immigrants as significantly warmer and perceived fewer social harms than those with other rationales.

No significant Rationale (less than human, virtuous violence, affect heuristic, recantations) X Measurement format (slider, clicking) interactions emerged for variables in S6 Table in S1 File, likely due to insufficient power. Only the AOM was manipulated, which also explains why interaction effects failed to obtain for the other measures.

## Discussion

The evidence from this study offers significant contributions to the dehumanization literature by suggesting why people dehumanize illegal immigrants and how rates of dehumanization are affected by measurement format (e.g., using a slider versus clicking on images of evolving hominids). In other work [7], nearly 38% of an online sample dehumanized illegal immigrants using the AOM measure via a slider. Clicking on unevolved human images perhaps encourages a more deliberate form of judgment than using a slider [27], a format that affected Republicans the most (Fig 2). Blatant dehumanization was substantially lower in the current study (18.6%; 125/672) and as low as 7.3% (49/672) if dominant themes were qualitatively identified relative to other rationales. People might also feel psychologically distant from their judgments using a slider, since clicking on a less-evolved figure forces participants to be resolute in their dislike for illegal immigrants. These findings are broadly consistent with survey methodology research suggesting scale features can impact judgments [36, 37].

The present results, including replications (e.g., dehumanization linked Republican ideology) and extensions of prior work (e.g., understanding why people dehumanize illegal immigrants), have broad significance beyond academic research. Chief among them is that they reveal dehumanization is often linked to factors other than a belief that the target is less than human. On the one hand, these data are reassuring because blatant dehumanization might be less pervasive than reported. On the other hand, they are still disturbing because of the many potential avenues that dehumanization might manifest, especially via covert forms of treating illegal immigrants as “less than.” For example, virtuous violence acknowledges the humanity of outgroups, but this rationale still degrades their worth in ways that suggest they deserve such harsh treatment. We also observed that a nontrivial number of people used their feelings when describing why they dehumanized. Affect as a rationale for dehumanization is also difficult to detect since emotions are often self-contained unless solicited. In light of these challenges, we encourage researchers and practitioners to evaluate how such forms of dehumanization might appear in other forms of behavior that might not require question-asking.

Our results suggest important pathways that people might dehumanize illegal immigrants and provide insights into why they think others might dehumanize as well. Automated text analyses revealed people often cite policy reasons for their dehumanization judgments, similar to how Republicans in the United States support their policy beliefs with articles from the Constitution [38]. Further, dislike was not significantly related to the AOM in our multiple regression model comparing the different reasons why people believe others might dehumanize illegal immigrants. This evidence is reasonable after considering models of affect and reasoning from moral psychology [35]. In the social intuitionist model, affect motivates one’s reasoning for a decision, though people often do not recognize that their response was driven by affect. Clearly, affect plays a role in dehumanization judgments but it is perhaps understated in this sample because it can be difficult to express, admit, or realize.

Our qualitative review of rationales revealed dehumanization is not a one-size-fits-all phenomenon where people universally perceive an outgroup as less than human. Virtuous violence, affect, and misunderstandings are important reasons why people make dehumanization judgments. These results matter because a nontrivial number of false-positives (e.g., recantations) might result from failing to identify rationales for less-than-human judgments. Here, we have learned nuances of the AOM and blatant dehumanization measurement.

In addition to the theoretical and methodological advancements of this article for dehumanization research, our work has a variety of implications for migrant and refugee studies as well. According to Hack-Polay and Igwe [39], migrant integration into a new society is complex, with individuals and families needing to find resources, safety, and security in unknown territories [40]. How people are treated during this integration process and beyond is critical for their well-being. The safety and security of migrants may be undermined if they face dehumanizing rhetoric (e.g., calling immigrants animals, foreign intruders [6]) or policies that make integration attempts difficult. Our work highlights the potential social and psychological roadblocks migrants might face to no fault of their own. For example, migrants might feel like others are less warm toward them and that they do not belong in a new society, which can produce negative downstream psychological consequences. Therefore, to ease the integration process for migrants and immigrations, organizations should acknowledge the ways that dehumanization might exist (e.g., how natives might treat migrants, opportunities that exist for natives but not migrants) and eliminate them from the integration process as best as possible.

### Limitations and future directions

While our work only evaluated dehumanization toward immigrants, we believe these results identify methodological considerations for dehumanization research in general, and reveal warning signs of those who treat others as “less than.” Prior work has called for a constellation of clues that indicate people who blatantly dehumanize [7]. This study suggests that replications and extensions are crucial for the development of a social, psychological, and demographic profile of dehumanizers. To prevent mass atrocities and the dehumanization of outgroups, we need empirical work to identify warning signs and reasons for blatant disregard of humanity.

We also encourage future research to evaluate how multiple presumed outgroups are dehumanized to investigate if rationales are consistent. Further, our evidence suggests dehumanization occurs in less than one out of every five participants. Oversampling might therefore be useful to ensure that the number of dehumanizers per study is large enough to obtain significant and meaningful effect sizes. Finally, in our examination of qualitative rationales, we searched for dominant themes since the texts were short and amenable to this approach. Longer texts might contain some thematic overlap and future work should evaluate this directly.

## Conclusion

Why people dehumanize and how dehumanization is measured matter. In this study, we provided evidence that rationales across participants are not consistent, though predictable by theory. For many people, dehumanization represents treating illegal immigrants as less than human, but for others, dehumanization is virtuous, motivated by affect, or difficult to appraise. Clicking on images of (un)evolved humans also curbs rates of dehumanization relative to using sliders. We encourage researchers and policymakers to evaluate dehumanization with respect to how it is measured and a diversity of possible theoretical perspectives.

## Supporting information

S1 File Supporting files—Contains all the supporting tables and figures. (DOCX)
