# Supplementary material for: Why we dehumanize illegal immigrants: A US mixed-methods study
Source: PLoS One. 2021 Oct 7;16(10):e0257912. doi: 10.1371/journal.pone.0257912 (PMC8496814; doi:10.1371/journal.pone.0257912)
Supplement: S1 File — (DOCX) [file pone.0257912.s001.docx]

**Expanded Measurement Information**

**Vulnerability Index Items**

The first four items were rated on a four-point scale from Strongly disagree to Strongly agree: (1) “I often feel discriminated against,” (2) “My whole world feels like it is falling apart,” (3) “People like me aren’t benefiting from the growth of the economy,” and (4) “I have very little control over the risks to my health.” The final two questions were rated on four-point scales from (1) Poor to (4) Excellent and reverse-scored for the creation of the index: (5) “Would you rate your personal health as excellent, good, fair, or poor?” and (6) “How would you rate the quality of medical care that is available to you and your family?” Scores on each item were averaged to create the index.

**Social Harms Index Questions**

(1) “What do you think is more important – to protect the right of Americans to own guns, OR to control gun ownership?” with two radio button responses (0 = control gun ownership, 1 = Protect the right of Americans to own guns)

(2) “Do you approve or disapprove of the current immigration raids that are being carried out across the country by federal immigration enforcement agents?” with two radio button responses (0 = disapprove, 1 = approve)

(3) “Do you favor or oppose the death penalty for persons convicted of murder?” with two radio button responses (0 = oppose, 1 = favor).

Supplementary Table S1

| Measure | *N* | *M* | *SD* | Min | Max |
| --- | --- | --- | --- | --- | --- |
| Jail time scale | 672 | 2.67 | 1.33 | 1.00 | 6.00 |
| Inclusion of the Other in the Self | 672 | 2.07 | 1.19 | 1.00 | 5.00 |
| Ascent of Man (%) | 672 | 93.00 | 17.00 | 12.50 | 100.00 |
| Adverse Childhood Experiences Scale | 672 | 2.07 | 2.26 | 0.00 | 10.00 |
| Vulnerability index | 667 | 2.06 | 0.55 | 1.00 | 4.00 |
| Harms index | 671 | 1.40 | 1.21 | 0.00 | 3.00 |
| Support the right to own guns | 671 | 0.44 | 0.50 | 0.00 | 1.00 |
| Support immigration raids | 672 | 0.43 | 0.50 | 0.00 | 1.00 |
| Support the death penalty for convicted murderers | 672 | 0.53 | 0.50 | 0.00 | 1.00 |
| Warmth | 668 | 50.35 | 32.59 | 0.00 | 100.00 |
| I dehumanize to teach immigrants a lesson | 125 | 5.18 | 1.81 | 1.00 | 7.00 |
| I feel bad when immigrants are punished | 125 | 3.03 | 1.83 | 1.00 | 7.00 |
| Immigrants are inferior to me | 125 | 3.63 | 1.90 | 1.00 | 7.00 |
| Immigrants are unfamiliar to me | 125 | 3.78 | 1.78 | 1.00 | 7.00 |
| Unhappiness index | 672 | 4.24 | 0.98 | 1.00 | 5.00 |
| Unhappiness: Family separation | 672 | 4.56 | 0.87 | 1.00 | 5.00 |
| Unhappiness: Discrimination | 672 | 4.09 | 1.14 | 1.00 | 5.00 |
| Unhappiness: Harsh conditions | 672 | 4.08 | 1.24 | 1.00 | 5.00 |

*Descriptive Statistics for Key Variables*

*Note.* Some participants, where *n* < 672, did not respond to particular items and some questions were only available to those who dehumanized (*n* = 125).

Supplementary Table S2

|  | Measure | 1 | 2 | 3 | 4 | 5 | 6 | 7 | 8 | 9 | 10 | 11 | 12 | 13 | 14 | 15 | 16 | 17 | 18 | 19 | 20 | 21 | 22 | 23 | 24 |
| --- | --- | --- | --- | --- | --- | --- | --- | --- | --- | --- | --- | --- | --- | --- | --- | --- | --- | --- | --- | --- | --- | --- | --- | --- | --- |
| 1 | Jail time scale | -- |  |  |  |  |  |  |  |  |  |  |  |  |  |  |  |  |  |  |  |  |  |  |  |
| 2 | IOS scale | -.349^**^ | -- |  |  |  |  |  |  |  |  |  |  |  |  |  |  |  |  |  |  |  |  |  |  |
| 3 | AOM | -.350^**^ | .236^**^ | -- |  |  |  |  |  |  |  |  |  |  |  |  |  |  |  |  |  |  |  |  |  |
| 4 | ACES | -.051 | .148^**^ | .051 | -- |  |  |  |  |  |  |  |  |  |  |  |  |  |  |  |  |  |  |  |  |
| 5 | Vulnerability scale | -.151^**^ | .188^**^ | .034 | .346^**^ | -- |  |  |  |  |  |  |  |  |  |  |  |  |  |  |  |  |  |  |  |
| 6 | Harms index | .536^**^ | -.444^**^ | -.296^**^ | -.099^*^ | -.241^**^ | -- |  |  |  |  |  |  |  |  |  |  |  |  |  |  |  |  |  |  |
| 7 | Support gun ownership | .401^**^ | -.340^**^ | -.192^**^ | -.043 | -.143^**^ | .824^**^ | -- |  |  |  |  |  |  |  |  |  |  |  |  |  |  |  |  |  |
| 8 | Support immigration raids | .482^**^ | -.416^**^ | -.331^**^ | -.151^**^ | -.276^**^ | .851^**^ | .619^**^ | -- |  |  |  |  |  |  |  |  |  |  |  |  |  |  |  |  |
| 9 | Support death penalty | .423^**^ | -.326^**^ | -.197^**^ | -.050 | -.169^**^ | .757^**^ | .386^**^ | .455^**^ | -- |  |  |  |  |  |  |  |  |  |  |  |  |  |  |  |
| 10 | Impersonal pronouns | .066 | .040 | -.013 | .054 | .021 | -.011 | -.057 | -.003 | .036 | -- |  |  |  |  |  |  |  |  |  |  |  |  |  |  |
| 11 | Emotion | -.064 | .062 | .020 | .022 | .029 | -.159^**^ | -.109^**^ | -.157^**^ | -.116^**^ | .069 | -- |  |  |  |  |  |  |  |  |  |  |  |  |  |
| 12 | Positive emotion | -.066 | -.007 | .029 | -.027 | .024 | -.103^**^ | -.083^*^ | -.099^*^ | -.065 | -.025 | .588^**^ | -- |  |  |  |  |  |  |  |  |  |  |  |  |
| 13 | Negative emotion | -.033 | .088^*^ | .008 | .042 | .019 | -.116^**^ | -.071 | -.121^**^ | -.087^*^ | .105^**^ | .776^**^ | -.043 | -- |  |  |  |  |  |  |  |  |  |  |  |
| 14 | Power words | .141^**^ | .015 | .019 | -.034 | -.041 | .115^**^ | .127^**^ | .100^**^ | .062 | -.055 | .239^**^ | .001 | .298^**^ | -- |  |  |  |  |  |  |  |  |  |  |
| 15 | Age | .064 | -.063 | -.077^*^ | -.025 | -.126^**^ | .187^**^ | .151^**^ | .193^**^ | .110^**^ | -.067 | -.173^**^ | -.066 | -.165^**^ | .008 | -- |  |  |  |  |  |  |  |  |  |
| 16 | Political orientation | .522^**^ | -.416^**^ | -.300^**^ | -.132^**^ | -.264^**^ | .784^**^ | .652^**^ | .717^**^ | .539^**^ | -.030 | -.177^**^ | -.137^**^ | -.114^**^ | .055 | .264^**^ | -- |  |  |  |  |  |  |  |  |
| 17 | Warmth | -.603^**^ | .593^**^ | .390^**^ | .138^**^ | .230^**^ | -.708^**^ | -.549^**^ | -.707^**^ | -.471^**^ | -.005 | .147^**^ | .105^**^ | .106^**^ | -.038 | -.204^**^ | -.668^**^ | -- |  |  |  |  |  |  |  |
| 18 | I dehumanize to teach immigrants a lesson | .571^**^ | -.402^**^ | -.432^**^ | -.064 | -.069 | .487^**^ | .286^**^ | .537^**^ | .202^*^ | .066 | .222^*^ | .124 | .186^*^ | .176^*^ | -.062 | .350^**^ | -.646^**^ | -- |  |  |  |  |  |  |
| 19 | I feel bad when immigrants are punished | -.553^**^ | .487^**^ | .405^**^ | .070 | .092 | -.456^**^ | -.283^**^ | -.532^**^ | -.140 | -.057 | -.130 | -.115 | -.075 | -.091 | -.016 | -.332^**^ | .666^**^ | -.702^**^ | -- |  |  |  |  |  |
| 20 | Immigrants are inferior to me | .364^**^ | -.268^**^ | -.435^**^ | .020 | -.044 | .401^**^ | .225^*^ | .435^**^ | .186^*^ | .031 | .118 | .151 | .028 | .061 | .134 | .342^**^ | -.495^**^ | .440^**^ | -.421^**^ | -- |  |  |  |  |
| 21 | Immigrants are unfamiliar to me | .000 | -.244^**^ | .096 | -160 | -.129 | .077 | -.041 | .104 | .112 | .167 | .159 | .131 | .109 | .185^*^ | -.031 | .159 | -.070 | .057 | .057 | .110 | -- |  |  |  |
| 22 | Unhappiness index | -.443^**^ | .359^**^ | .360^**^ | .084^*^ | .135^**^ | -.550^**^ | -.462^**^ | -.522^**^ | -.355^**^ | -.010 | .060 | .039 | .049 | -.005 | -.152^**^ | -.537^**^ | .545^**^ | -.327^**^ | .385^**^ | -.270^**^ | .036 | -- |  |  |
| 23 | Unhappiness: Family separation | -.369^**^ | .276^**^ | .368^**^ | .049 | .054 | -.412^**^ | -.344^**^ | -.404^**^ | -.257^**^ | -.015 | .053 | .018 | .060 | .020 | -.106^**^ | -.414^**^ | .441^**^ | -.202^*^ | .314^**^ | -.276^**^ | .076 | .842^**^ | -- |  |
| 24 | Unhappiness: Discrimination | -.398^**^ | .331^**^ | .303^**^ | .120^**^ | .160^**^ | -.498^**^ | -.420^**^ | -.470^**^ | -.324^**^ | .006 | .024 | .034 | .009 | -.034 | -.135^**^ | -.487^**^ | .500^**^ | -.349^**^ | .357^**^ | -.256^**^ | -.029 | .923^**^ | .674^**^ | -- |
| 25 | Unhappiness: Harsh conditions | -.425^**^ | .353^**^ | .316^**^ | .055 | .134^**^ | -.556^**^ | -.468^**^ | -.522^**^ | -.363^**^ | -.018 | .083^*^ | .048 | .065 | .005 | -.162^**^ | -.534^**^ | .522^**^ | -.326^**^ | .370^**^ | -.209^*^ | .051 | .930^**^ | .674^**^ | .797^**^ |

*Bivariate Correlation Matrix* *Across Conditions*

*Note*. ***p* < .01, * *p* < .05. Political orientation is a 7-point scale from (1 = extremely liberal, to 7 extremely conservative). IOS = Inclusion of the Other in the Self, AOM = Ascent of Man, ACES = Adverse Childhood Experiences Scale.

Supplementary Table S3

|  | Measure | 1 | 2 | 3 | 4 | 5 | 6 | 7 | 8 | 9 | 10 | 11 | 12 | 13 | 14 | 15 | 16 | 17 | 18 | 19 | 20 | 21 | 22 | 23 | 24 |
| --- | --- | --- | --- | --- | --- | --- | --- | --- | --- | --- | --- | --- | --- | --- | --- | --- | --- | --- | --- | --- | --- | --- | --- | --- | --- |
| 1 | Jail time scale | -- |  |  |  |  |  |  |  |  |  |  |  |  |  |  |  |  |  |  |  |  |  |  |  |
| 2 | IOS scale | -.416^**^ | -- |  |  |  |  |  |  |  |  |  |  |  |  |  |  |  |  |  |  |  |  |  |  |
| 3 | AOM | -.320^**^ | .247^**^ | -- |  |  |  |  |  |  |  |  |  |  |  |  |  |  |  |  |  |  |  |  |  |
| 4 | ACES | -.050 | .181^**^ | .125^*^ | -- |  |  |  |  |  |  |  |  |  |  |  |  |  |  |  |  |  |  |  |  |
| 5 | Vulnerability scale | -.128^*^ | .205^**^ | -.024 | .381^**^ | -- |  |  |  |  |  |  |  |  |  |  |  |  |  |  |  |  |  |  |  |
| 6 | Harms index | .596^**^ | -.468^**^ | -.276^**^ | -.057 | -.250^**^ | -- |  |  |  |  |  |  |  |  |  |  |  |  |  |  |  |  |  |  |
| 7 | Support gun ownership | .477^**^ | -.366^**^ | -.199^**^ | -.022 | -.150^**^ | .843^**^ | -- |  |  |  |  |  |  |  |  |  |  |  |  |  |  |  |  |  |
| 8 | Support immigration raids | .543^**^ | -.420^**^ | -.304^**^ | -.124^*^ | -.291^**^ | .850^**^ | .626^**^ | -- |  |  |  |  |  |  |  |  |  |  |  |  |  |  |  |  |
| 9 | Support death penalty | .461^**^ | -.377^**^ | -.179^**^ | .000 | -.180^**^ | .786^**^ | .462^**^ | .484^**^ | -- |  |  |  |  |  |  |  |  |  |  |  |  |  |  |  |
| 10 | Impersonal pronouns | .007 | .058 | .039 | .101 | .065 | -.036 | -.055 | -.050 | .021 | -- |  |  |  |  |  |  |  |  |  |  |  |  |  |  |
| 11 | Emotion | -.075 | .011 | .017 | -.035 | .003 | -.154^**^ | -.082 | -.153^**^ | -.141^**^ | .012 | -- |  |  |  |  |  |  |  |  |  |  |  |  |  |
| 12 | Positive emotion | -.029 | -.031 | .041 | -.071 | -.007 | -.113^*^ | -.085 | -.139^*^ | -.051 | -.045 | .574^**^ | -- |  |  |  |  |  |  |  |  |  |  |  |  |
| 13 | Negative emotion | -.076 | .040 | -.014 | .007 | .014 | -.106 | -.041 | -.086 | -.133^*^ | .046 | .777^**^ | -.062 | -- |  |  |  |  |  |  |  |  |  |  |  |
| 14 | Power words | .165^**^ | -.024 | -.018 | -.005 | -.078 | .139^*^ | .174^**^ | .109^*^ | .077 | -.108^*^ | .193^**^ | -.027 | .260^**^ | -- |  |  |  |  |  |  |  |  |  |  |
| 15 | Age | .124^*^ | -.081 | -.154^**^ | -.072 | -.098 | .224^**^ | .137^*^ | .240^**^ | .179^**^ | -.047 | -.127^*^ | -.043 | -.123^*^ | .086 | -- |  |  |  |  |  |  |  |  |  |
| 16 | Political orientation | .556^**^ | -.444^**^ | -.212^**^ | -.114^*^ | -.251^**^ | .807^**^ | .675^**^ | .720^**^ | .606^**^ | -.035 | -.145^**^ | -.128^*^ | -.082 | .099 | .265^**^ | -- |  |  |  |  |  |  |  |  |
| 17 | Warmth | -.672^**^ | .607^**^ | .341^**^ | .171^**^ | .242^**^ | -.720^**^ | -.572^**^ | -.707^**^ | -.508^**^ | .025 | .134^*^ | .075 | .111^*^ | -.071 | -.235^**^ | -.682^**^ | -- |  |  |  |  |  |  |  |
| 18 | I dehumanize to teach immigrants a lesson | .484^**^ | -.399^**^ | -.243 | .048 | -.105 | .149 | .062 | .270 | -.038 | -.033 | .261 | .085 | .252 | .137 | -.083 | .145 | -.570^**^ | -- |  |  |  |  |  |  |
| 19 | I feel bad when immigrants are punished | -.614^**^ | .419^**^ | .252 | .110 | .124 | -.245 | -.143 | -.353^*^ | .021 | .091 | -.146 | -.159 | -.072 | .052 | -.110 | -.126 | .585^**^ | -.571^**^ | -- |  |  |  |  |  |
| 20 | Immigrants are inferior to me | .334^*^ | -.072 | -.420^**^ | -.024 | .060 | .249 | .165 | .291 | .022 | -.234 | .120 | .037 | .115 | -.046 | .238 | .322^*^ | -.304 | .150 | -.257 | -- |  |  |  |  |
| 21 | Immigrants are unfamiliar to me | -.018 | -.266 | .061 | -.090 | -.131 | -.017 | -.198 | .067 | .145 | .231 | .219 | .163 | .179 | .252 | -.079 | .193 | -.127 | .166 | .259 | .030 | -- |  |  |  |
| 22 | Unhappiness index | -.511^**^ | .368^**^ | .405^**^ | .099 | .138^*^ | -.590^**^ | -.473^**^ | -.561^**^ | -.433^**^ | -.011 | .002 | .026 | -.020 | -.070 | -.205^**^ | -.567^**^ | .585^**^ | -.346^*^ | .420^**^ | -.418^**^ | -.029 | -- |  |  |
| 23 | Unhappiness: Family separation | -.426^**^ | .298^**^ | .410^**^ | .072 | .032 | -.469^**^ | -.373^**^ | -.453^**^ | -.341^**^ | -.012 | .010 | .007 | .005 | -.086 | -.181^**^ | -.457^**^ | .487^**^ | -.261 | .442^**^ | -.497^**^ | .135 | .846^**^ | -- |  |
| 24 | Unhappiness: Discrimination | -.427^**^ | .351^**^ | .352^**^ | .145^**^ | .176^**^ | -.513^**^ | -.412^**^ | -.483^**^ | -.380^**^ | .004 | -.044 | .025 | -.073 | -.049 | -.184^**^ | -.519^**^ | .523^**^ | -.365^*^ | .338^*^ | -.311^*^ | -.086 | .920^**^ | .676^**^ | -- |
| 25 | Unhappiness: Harsh conditions | -.518^**^ | .340^**^ | .350^**^ | .051 | .142^**^ | -.598^**^ | -.480^**^ | -.567^**^ | -.437^**^ | -.020 | .038 | .034 | .016 | -.060 | -.189^**^ | -.546^**^ | .563^**^ | -.308^*^ | .364^*^ | -.337^*^ | -.107 | .932^**^ | .684^**^ | .790^**^ |

*Bivariate Correlation Matrix* *for Clicking Condition*

*Note*. ***p* < .01, * *p* < .05. Political orientation is a 7-point scale from (1 = extremely liberal, to 7 extremely conservative). IOS = Inclusion of the Other in the Self, AOM = Ascent of Man, ACES = Adverse Childhood Experiences Scale.

Supplementary Table S4

|  | Measure | 1 | 2 | 3 | 4 | 5 | 6 | 7 | 8 | 9 | 10 | 11 | 12 | 13 | 14 | 15 | 16 | 17 | 18 | 19 | 20 | 21 | 22 | 23 | 24 |
| --- | --- | --- | --- | --- | --- | --- | --- | --- | --- | --- | --- | --- | --- | --- | --- | --- | --- | --- | --- | --- | --- | --- | --- | --- | --- |
| 1 | Jail time scale | -- |  |  |  |  |  |  |  |  |  |  |  |  |  |  |  |  |  |  |  |  |  |  |  |
| 2 | IOS scale | -.281^**^ | -- |  |  |  |  |  |  |  |  |  |  |  |  |  |  |  |  |  |  |  |  |  |  |
| 3 | AOM | -.391^**^ | .229^**^ | -- |  |  |  |  |  |  |  |  |  |  |  |  |  |  |  |  |  |  |  |  |  |
| 4 | ACES | -.053 | .111^*^ | -.003 | -- |  |  |  |  |  |  |  |  |  |  |  |  |  |  |  |  |  |  |  |  |
| 5 | Vulnerability scale | -.178^**^ | .168^**^ | .075 | .307^**^ | -- |  |  |  |  |  |  |  |  |  |  |  |  |  |  |  |  |  |  |  |
| 6 | Harms index | .474^**^ | -.420^**^ | -.326^**^ | -.146^*^ | -.231^**^ | -- |  |  |  |  |  |  |  |  |  |  |  |  |  |  |  |  |  |  |
| 7 | Support gun ownership | .326^**^ | -.312^**^ | -.193^**^ | -.065 | -.134^*^ | .804^**^ | -- |  |  |  |  |  |  |  |  |  |  |  |  |  |  |  |  |  |
| 8 | Support immigration raids | .424^**^ | -.410^**^ | -.355^**^ | -.179^**^ | -.259^**^ | .855^**^ | .612^**^ | -- |  |  |  |  |  |  |  |  |  |  |  |  |  |  |  |  |
| 9 | Support death penalty | .383^**^ | -.281^**^ | -.230^**^ | -.104 | -.160^**^ | .730^**^ | .311^**^ | .431^**^ | -- |  |  |  |  |  |  |  |  |  |  |  |  |  |  |  |
| 10 | Impersonal pronouns | .127^*^ | .015 | -.066 | .001 | -.032 | .017 | -.058 | .050 | .047 | -- |  |  |  |  |  |  |  |  |  |  |  |  |  |  |
| 11 | Emotion | -.056 | .111^*^ | .012 | .082 | .055 | -.163^**^ | -.135^*^ | -.159^**^ | -.096 | .125^*^ | -- |  |  |  |  |  |  |  |  |  |  |  |  |  |
| 12 | Positive emotion | -.106 | .014 | .013 | .019 | .058 | -.092 | -.080 | -.056 | -.084 | -.009 | .600^**^ | -- |  |  |  |  |  |  |  |  |  |  |  |  |
| 13 | Negative emotion | .009 | .136^*^ | .018 | .079 | .023 | -.126^*^ | -.102 | -.156^**^ | -.044 | .166^**^ | .776^**^ | -.025 | -- |  |  |  |  |  |  |  |  |  |  |  |
| 14 | Power words | .117^*^ | .056 | .047 | -.066 | .001 | .090 | .079 | .091 | .046 | .003 | .288^**^ | .032 | .339^**^ | -- |  |  |  |  |  |  |  |  |  |  |
| 15 | Age | .007 | -.047 | -.028 | .021 | -.155^**^ | .150^**^ | .165^**^ | .148^**^ | .045 | -.089 | -.219^**^ | -.088 | -.206^**^ | -.069 | -- |  |  |  |  |  |  |  |  |  |
| 16 | Political orientation | .488^**^ | -.386^**^ | -.372^**^ | -.152^**^ | -.278^**^ | .761^**^ | .630^**^ | .714^**^ | .474^**^ | -.023 | -.209^**^ | -.146^**^ | -.147^**^ | .009 | .264^**^ | -- |  |  |  |  |  |  |  |  |
| 17 | Warmth | -.530^**^ | .575^**^ | .446^**^ | .100 | .216^**^ | -.696^**^ | -.526^**^ | -.707^**^ | -.435^**^ | -.042 | .160^**^ | .135^*^ | .100 | .000 | -.174^**^ | -.653^**^ | -- |  |  |  |  |  |  |  |
| 18 | I dehumanize to teach immigrants a lesson | .584^**^ | -.390^**^ | -.511^**^ | -.089 | -.079 | .576^**^ | .353^**^ | .611^**^ | .263^*^ | .105 | .212 | .155 | .148 | .175 | -.076 | .453^**^ | -.661^**^ | -- |  |  |  |  |  |  |
| 19 | I feel bad when immigrants are punished | -.542^**^ | .520^**^ | .476^**^ | .044 | .084 | -.543^**^ | -.344^**^ | -.608^**^ | -.202 | -.141 | -.121 | -.100 | -.074 | -.157 | .040 | -.444^**^ | .711^**^ | -.762^**^ | -- |  |  |  |  |  |
| 20 | Immigrants are inferior to me | .374^**^ | -.325^**^ | -.444^**^ | .027 | -.102 | .454^**^ | .244^*^ | .486^**^ | .241^*^ | .171 | .118 | .203 | -.027 | .103 | .077 | .355^**^ | -.565^**^ | .547^**^ | -.495^**^ | -- |  |  |  |  |
| 21 | Immigrants are unfamiliar to me | .007 | -.245^*^ | .111 | -.194 | -.130 | .115 | .029 | .121 | .100 | .132 | .125 | .118 | .067 | .153 | -.007 | .142 | -.048 | .014 | -.043 | .147 | -- |  |  |  |
| 22 | Unhappiness index | -.376^**^ | .350^**^ | .341^**^ | .069 | .131^*^ | -.508^**^ | -.451^**^ | -.484^**^ | -.279^**^ | -.010 | .118^*^ | .051 | .118^*^ | .062 | -.102 | -.507^**^ | .502^**^ | -.298^**^ | .366^**^ | -.197 | .068 | -- |  |  |
| 23 | Unhappiness: Family separation | -.314^**^ | .254^**^ | .355^**^ | .025 | .078 | -.355^**^ | -.316^**^ | -.357^**^ | -.175^**^ | -.017 | .095 | .030 | .115^*^ | .127^*^ | -.035 | -.373^**^ | .395^**^ | -.159 | .246^*^ | -.170 | .048 | .839^**^ | -- |  |
| 24 | Unhappiness: Discrimination | -.370^**^ | .311^**^ | .281^**^ | .095 | .144^**^ | -.483^**^ | -.427^**^ | -.458^**^ | -.270^**^ | .007 | .091 | .043 | .092 | -.019 | -.088 | -.456^**^ | .476^**^ | -.322^**^ | .365^**^ | -.224^*^ | -.002 | .927^**^ | .672^**^ | -- |
| 25 | Unhappiness: Harsh conditions | -.330^**^ | .365^**^ | .301^**^ | .058 | .125^*^ | -.512^**^ | -.456^**^ | -.476^**^ | -.291^**^ | -.018 | .128^*^ | .061 | .114^*^ | .076 | -.136^*^ | -.521^**^ | .476^**^ | -.314^**^ | .372^**^ | -.143 | .130 | .929^**^ | .664^**^ | .804^**^ |

*Bivariate Correlation Matrix* *for Slider Condition*

*Note*. ***p* < .01, * *p* < .05. Political orientation is a 7-point scale from (1 = extremely liberal, to 7 extremely conservative). IOS = Inclusion of the Other in the Self, AOM = Ascent of Man, ACES = Adverse Childhood Experiences Scale.

Supplementary Table S5

*Results from Varimax Rotated Principal Component Analysis*

| C1 | | C2 | | C3 | | C4 | | C5 | |
| --- | --- | --- | --- | --- | --- | --- | --- | --- | --- |
| Law breaking | | Criminality | | Entering illegally | | Rights like Americans | | Decision-making process | |
| λ | % | λ | % | Λ | % | λ | % | λ | % |
| 3.75 | 5.69 | 3.41 | 5.16 | 3.12 | 4.73 | 2.88 | 4.36 | 2.71 | 4.10 |
| Word | Loading | Word | Loading | Word | Loading | Word | Loading | Word | Loading |
| follow | 0.656 | commit | 0.644 | cross | 0.857 | rate | 0.608 | advance | 0.630 |
| law | 0.605 | basic | 0.597 | border | 0.811 | immigrant | 0.552 | reason | 0.600 |
| break | 0.597 | crime | 0.591 | illegally | 0.495 | deserve | 0.486 | choose | 0.475 |
| punish | 0.590 | right | 0.538 | enter | 0.448 | american | 0.482 | lower | 0.464 |
| society | 0.573 | life | 0.529 | legally | 0.432 | illegal | 0.474 | country | 0.461 |
| civilize | 0.445 | rule | 0.494 | people | 0.413 | general | 0.399 | develop | 0.456 |
| place | 0.444 | wrong | 0.418 | lack | 0.325 | realize | 0.388 | consider | 0.304 |
| state | 0.371 | live | 0.300 | moral | 0.318 | family | 0.382 |  |  |
| america | 0.331 |  |  |  |  | question | 0.373 |  |  |
|  |  |  |  |  |  | legal | 0.343 |  |  |

*Note*. C1-C5 = Components 1 through 5.

Supplementary Table S6

|  | Less than human  (*n* = 49) | | Virtuous Violence  (*n* = 35) | | Affect Heuristic  (*n* = 17) | | Recantations  (*n* = 22) | |  |  |  |
| --- | --- | --- | --- | --- | --- | --- | --- | --- | --- | --- | --- |
|  | *M* | *SE* | *M* | *SE* | *M* | *SE* | *M* | *SE* | *F* | *p* | *η*^2^*_p_* |
| Jail time scale | 3.80^a^ | 0.17 | 3.60^a^ | 0.20 | 3.65^a^ | 0.29 | 2.55^b^ | 0.25 | 6.00 | .001 | .131 |
| AOM (%) | 59.20^a^ | 3.10 | 72.80^b^ | 3.60 | 55.10^a^ | 5.20 | 74.90^b^ | 4.60 | 5.51 | .001 | .122 |
| IOS scale | 1.16^a^ | 0.11 | 1.51^a,b^ | 0.13 | 1.53^a,b^ | 0.19 | 1.91^b^ | 0.17 | 4.81 | .003 | .108 |
| Warmth | 13.47^a,†^ | 3.41 | 26.97^a,†^ | 4.03 | 24.13^a^ | 5.97 | 49.91^b^ | 5.09 | 11.88 | < .001 | .232 |
| Harms index | 2.59^a,†^ | 0.12 | 2.14^a,†^ | 0.14 | 2.24^a^ | 0.20 | 1.46^b^ | 0.18 | 9.54 | < .001 | .194 |
| Unhappiness index | 3.14^a,†^ | 0.16 | 3.79^a,b,†^ | 0.19 | 3.37^a,b^ | 0.28 | 3.94^b^ | 0.24 | 3.61 | .015 | .083 |

*Statistical Effects Based on Qualitative Coding Results*

*Note*. Different superscripts indicate *Bonferroni*-corrected differences at *p* < .05. ^†^ = two rationales are significantly different at *p* < .10.

Supplementary Figure S1

*Hotspot Feature Using the Ascent of Man Scale*


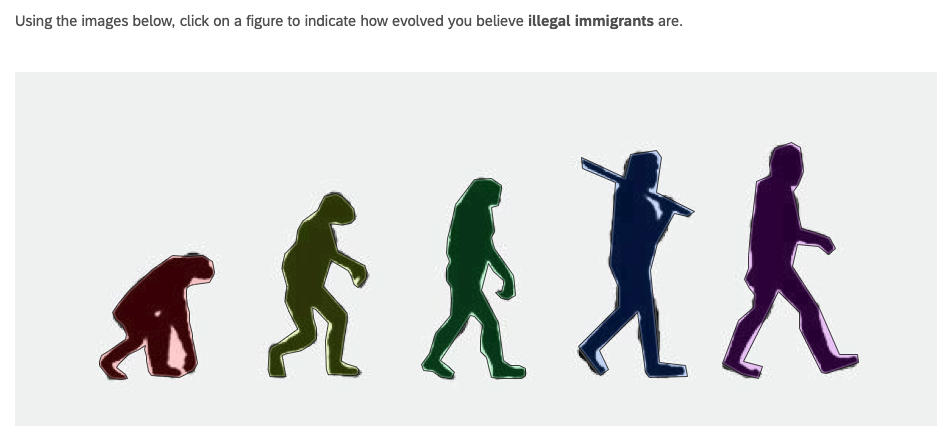


Qualitative Analysis Codebook

(1) Less than human: people genuinely believe that immigrants are less evolved, less than human, and some aspect of their humanity is “less than” (e.g., they're dirty, they're animals, they're less intelligent for breaking the law etc.) relative to Americans or other groups. If participants do not refute the idea of an illegal immigrant being less than human, this is likely the appropriate choice. This excludes personal experience.

(2) Virtuous violence: people acknowledge that illegal immigrants are human, but they justify dehumanization by inferiority on other characteristics (e.g., intelligence, morals). Such explanations can be used to justify violence and harsh treatment. Therefore, if people believe that immigrants are human (this is clearly stated in the text) but dehumanize them for other reasons (e.g., they lack intelligence, morals) this is likely the appropriate choice.

Responses stating “I did not mean to rate illegal immigrants as less than human, but [insert justification here]...” would be appropriate for virtuous violence and not a recantation.

(3) Affect heuristic: people rely on feelings and emotions to judge how they perceive illegal immigrants. If people rely on their feeling system (emotions) to suggest an immigrant is bad or unworthy of being called a human, this would be in support of the affect heuristic hypothesis. If people suggest that they just don't like immigrants, this is likely the appropriate choice. If people had a personally negative experience with immigrants and this informed their decision, this is likely the appropriate choice.

(4) Recantations: anytime a participant went back on their rating and suggested that it was a mistake, they didn't understand what the figure meant, or if they gave the same rating to Americans as well, this is likely the appropriate choice.

Any time that a participant justifies a form of dehumanization or violence, while suggesting they did not mean to call immigrants less evolved, this is not a recantation. They are still justifying dehumanization and recognizing the illegal immigrants’ humanity at the same time.
